# Association between *Helicobacter pylori* Infection and Pancreatic Cancer Development: A Meta-Analysis

**DOI:** 10.1371/journal.pone.0075559

**Published:** 2013-09-26

**Authors:** Mingjia Xiao, Yiming Wang, Yi Gao

**Affiliations:** 1 Department of Hepatobiliary Surgery, Wuxi People’s Hospital of Nanjing Medical University, Wuxi, Jiangsu Province, China; 2 Second Department of Hepatobiliary Surgery, Zhujiang Hospital of Southern Medical University, Guangzhou, Guangdong Province, China; 3 Department of Urology Surgery, Zhujiang Hospital of Southern Medical University, Guangzhou, Guangdong Province, China; Tongji Medical College, Huazhong University of Science and Technology, China

## Abstract

**Background:**

Pancreatic cancer is one of the most troublesome malignancies with dismal prognosis. *H. pylori* has been recognized as a type I carcinogen. Several studies have evaluated the association between *H. pylori* infection and pancreatic cancer development, however, the conclusions are inconsistent.

**Methods:**

Literature search was carried out in PubMed, EMBASE, Cochrane Library and CNKI databases to identify eligible researches. We performed overall meta-analysis of all studies included and subgroup analysis based on regional distribution. Quality of the studies (assessed by Newcastle-Ottawa quality assessment scale for case-control studies) and CagA+ strains of *H. pylori* were taken into consideration, and we conducted additional analyses including high-quality researches and those concerning CagA+ *H. pylori* respectively.

**Results:**

9 studies involving 3033 subjects (1083 pancreatic cancer cases, 1950 controls) were included. Summary OR and 95%CI of the overall meta-analysis of all included studies were 1.47 and 1.22-1.77, pooled data of the 4 high-quality studies were OR 1.28, 95%CI 1.01-1.63. OR of the 5 studies examined CagA+ strains was 1.42, corresponding 95%CI was 0.79 to 2.57. Summary estimates of subgroup analysis based on regional distribution are as follows, Europe group: OR 1.56, 95%CI 1.15-2.10; East Asia group: OR 2.01, 95%CI 1.33-3.02; North America group: OR 1.17, 95%CI 0.87-1.58. There was not obvious heterogeneity across the 9 studies. No publication bias was detected.

**Conclusion:**

*H. pylori* infection is significantly, albeit weakly, associated with pancreatic cancer development. The association is prominent in Europe and East Asia, but not in North America. CagA+ *H. pylori* strains appear not to be associated with pancreatic cancer. However, more studies, especially prospective studies, are needed to validate our results.

## Introduction

Pancreatic cancer, also known as exocrine pancreatic carcinoma or pancreatic ductal adenocarcinoma [1], is the fifth leading cause of cancer related death worldwide [2] and the fourth in the USA [3], due to the advanced stage at diagnosis and poor responses to current treatments [4]. Despite steadily increasing understandings on the mechanisms underlying carcinogenesis of pancreatic cancer, there is still a long way to go to apply the advanced knowledge to the clinical practice to get an ideal solution for this troublesome malignancy. Previous epidemiological investigations have identified some possible risk factors of pancreatic cancer, such as smoking, chronic pancreatitis, long-standing diabetes, mutations of various genes and so on [5-7]. However, preventive strategies taking these risk factors into account appear not to achieve what expected. Therefore, identification of more potential risk factors leading to effective primary prevention is still in great urgency.

Since Marshall and Warren reported in 1984 [8] a curved bacillus that may serve as the causal factor of gastritis and peptic ulceration, a strong link has been established between *Helicobacter pylori* especially the CagA (a product of cytotoxin associated gene A) positive strains, and a diverse spectrum of diseases, such as, acute and chronic gastritis, peptic ulcer disease, gastric cancer, mucosa-associated lymphoid tissue (MALT) lymphoma [9]. In 1994, *H. pylori* was classified as a type I (definite) carcinogen of stomach cancer by World Health Organization (WHO) [10]. The prevalence of *H. pylori* infection ranges from over 70% in the developing countries to less than 40% in the developed world [11,12]. Three interesting phenomena are worth particular notice. Firstly, only a small portion of carriers of *H. pylori* infection develop tumor related to its presence [13]. Secondly, some studies have suggested an inverse association between *H. pylori* infection and gastroesophageal reflux disease (GERD) [14] or esophageal adenocarcinoma [15]. Thirdly, it has been reported that *H. pylori* infection may be associated with some extra-gastrointestinal diseases, such as cholelithiasis [16], coronary heart disease [17], childhood asthma [18] and so on. All these findings imply that the interactions between *H. pylori* and humans are extremely sophisticated but not fully understood.

Several studies evaluating the possible association between *H. pylori* infection and pancreatic cancer development have been published to date [19-27]. However, the conclusions are not unanimous, which may be attributed to three major factors. First, the sample sizes are generally small, with than 300 patients in most studies. Insufficient samples could enormously weaken the power to identify the possible association and even lead to false conclusions. Second, studies carried out in different regions have produced controversial results, for example, studies in China demonstrated a strong association between *H. pylori* infection and pancreatic cancer [22,23] whereas those in Europe did not [21,24]. Third, all of these studies are case-control studies or nested case-control studies, the quality of which is inconsistent. It has been shown that case control studies are easily biased compared to prospective clinical trials [28]. Meta-analysis seems to be a promising method to detect the potential association as a convincing conclusion should not be drawn from any individual studies. Two meta-analyses were published in 2011 and 2012, respectively, both favoring the positive link between *H. pylori* infection and pancreatic cancer development [25,29]. However, three obvious short comings in these two meta-analyses. First, they did not assess the influence of geographic or racial factors nor consider the other confounding risk factors. Second, they did not taken the quality of the studies into account. Third, they did not address the status of CagA. Moreover, more new studies on this topic have been published since these meta-analyses, still with inconsistent conclusions [22,24,26,27].

Based on current understanding of pancreatic cancer and *H. pylori*, we endeavored to conduct this updated meta-analysis to further investigate the possible association, by trying our best to collect all eligible studies up to May 2013, taking the quality of the studies, the CagA-positive strains of *H. pylori* and geographic regions and races into account.

## Materials and Methods

This meta-analysis was performed following QUOROM guidelines [30]. The procedures adhered to the PRISMA Statement guidelines (Checklist S1).

### Literature search

Studies were identified by a systematic literature search in PubMed, EMBASE, Cochrane Library and CNKI (China National Knowledge Infrastructure) databases from inception to May, 2013. The searching strategy was decided by discussions among all authors and improved in several turns of attempts to get results as comprehensive as possible, involving terms in 3 key aspects: (1) *H. pylori*: “ *Helicobacter* ”, “*Helicobacter Pylori*”, “Hp”, “*H. pylori*”, “ *Helicobacter* species”, “ *Helicobacter* sp.”, “Helicobacter genus”. (2) pancreas: “pancreatic”, “pancreas”. (3) cancer: “cancer”, “tumor”, “tumour”, “neoplasm”, “malignancy”, “adenocarcinoma”, “carcinoma”. Both Mesh subjects and keywords were applied during PubMed search, with the filter activated of “humans”. Searching terms were checked and adapted by Emtree terms when using EMBASE. We imposed no language restriction. In addition, a manual search of citations of the included studies was performed.

### Study selection

Two investigators (Mingjia Xiao & Yiming Wang) independently selected the studies included into the meta-analysis. Disagreements were resolved by discussion or consultation to the corresponding author (Yi Gao).

We selected the studies for inclusion if they fulfilled the following criteria: (1) The study should concern the association between *H. pylori* infection and pancreatic cancer development; (2) The study must be conducted in adults, defined as patients greater than 18 years old; (3) There must be a control group; (4) *H. pylori* infection with or without status of CagA; should be determined by serological testing (like ELISA, Western blotting) or any other reliable method; (5) The study must provide the sample size and data on the positive rates of *H. pylori* infection between the groups with odds ratios (OR) and the corresponding 95% confidence interval (95%CI); (6) Diagnoses of the patients with and without pancreatic cancer (exocrine pancreatic carcinoma, pancreatic ductal carcinoma) must be confirmed by pathological examination; and (7) The research was approved by the Ethics Committee.

### Data extraction

The two investigators extracted the data independently using a unified data form and the results were cross-checked. In the case of discrepancy, decisions were made by discussion. The data retrieved covered first author, publication year, country, study design, sample size, method for detecting *H. pylori*, number of *H. pylori* positive cases in both groups, OR and corresponding 95%CI of pancreatic cancer for *H. pylori* infection, and adjustments. If there was an adjusted OR with 95%CI by other risk factors, such as age, gender, *etc.*, reported in the paper, we recorded the adjusted data; if not, the crude data were recorded and used instead.

### Quality assessment of the included research

Investigators assessed the methodological quality of every included study by Newcastle-Ottawa quality assessment scale (NOS) for case-control studies, which contains 8 items (1 star for each, and up to 8 stars in total) categorized into 3 major categories: (1) Selection: adequate definition of cases, representativeness of the cases, definition of controls; (2) Comparability: the controls were matched to cases on age and sex, controls were matched by other confounding factors; (3) Exposure: ascertainment of exposures, the same methods to ascertain the exposure for cases and controls, the same non-response rate in both groups [31]. The ultimate score of 6 stars or more was regarded as high-quality.

### Statistical analysis

Overall meta-analysis of all included studies was carried out to determine the association of *H. pylori* infection and pancreatic cancer risk. In addition, three more sub-analyses were conducted (1), meta-analysis including only the high quality researches (2), meta-analysis evaluating carcinogenetic risk of infection with CagA+ *H. pylori* strains, and (3) subgroup analysis according to geographic distribution of the studies.

Cumulative OR and corresponding 95%CI were calculated as the summary estimates to measure the strength of the associations. The heterogeneity across the studies was examined by *χ* ^*2*^-based *I* ^2^ test and considered significant if *I* ^2^≥50%. In the case of significant heterogeneity, a meta-analysis was performed using a random effect model by the DerSimonian and Laird method [32]; otherwise a fixed effect model and the Mantel–Haenszel weighting algorithm [33] were preferred. In order to exclude the possible influence of any single research, sensitivity analysis was performed to evaluate whether omitting one study in turn substantially altered the results or magnitude of the summary estimates of the remainders.

Visual inspection of funnel plots, Begg’s rank correlation test [34] and Egger’s regression test [35] were all generated to detect potential publication bias. All *P* values were set two sided, and those less than 0.05 were deemed statistically significant. Our meta-analysis was performed by using Stata 12.0 and RevMan 5.1 software.

## Results

### Characteristics of included studies

**Figure 1:**
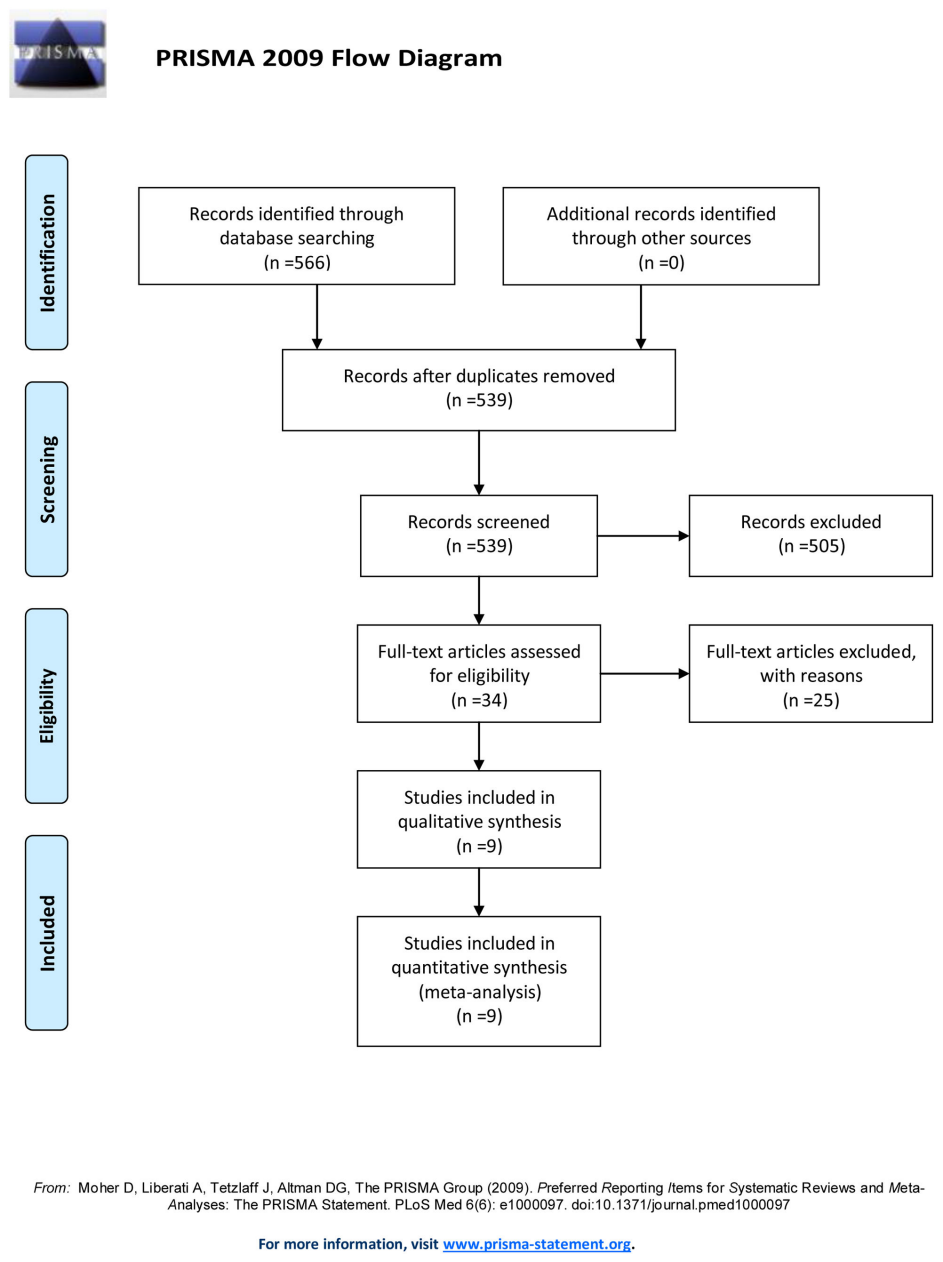
Flow diagram of literature search and study selection.

Systematic search yielded a total of 566 papers. After reviewing titles, abstracts and full texts, nine studies involving 3033 subjects (1083 pancreatic cancer cases and 1950 controls) were finally retrieved based on the pre-defined inclusion and exclusion criteria [19-24,36-38]. A diagram schematizing the selection process is presented in Figure 1. All selected studies are case-control studies, including three nested-control studies [20,21,36]. The main characteristics of included studies are summarized in Table 1. Among them, four were from Europe (Austria, Finland, Sweden and Poland) [19-21,24], 2 from North America (the USA) [36,37] and three from East Asia (Japan and China) [22,23,38]. All studies used serological method, ELISA or Western blotting to detect antibodies to *H. pylori* whole cell proteins to identify *H. pylori* infection. Six studies [20,22-24,36,37] went further to detect antibodies toCagA protein of *H. pylori*, and one of them detected CagA and VacA protein [23]. Age, sex, cigarette smoking or other possible confounding factors were matched between the cases and controls in four studies and ORs with 95% CIs were adjusted by Logistic regression [20,21,36,37], whereas the other five studies did not use controls individually matched to the cases.

**Table 1:** Characteristics of the 9 included studies.

Author	Year,country	Study design	Method	*H. pylori*(+) in cancer group	*H. pylori*(+) in control group	Cag+ in cancer group	Cag+ in control group	OR(95%CI)	Adjustments^b^
Raderer et al.	1997, Austria	Case-control	ELISA	60/92	28/62^a^	-	-	*H. pylori*: 2.1 (1.1, 4.1)	Not reported
Stolzenberg et al.	2001, Finland	Nested case-control	ELISA	99/121	165/226	73/121	115/226	*H. pylori*: 1.87 (1.05, 3.34); CagA: 2.01 (1.09, 3.70)	(1)(2)(3)(4)(5)
de Martel et al.	2008, the USA	Nested case-control	ELISA	51/104	155/262	33/104	83/262	*H. pylori*: 0.85 (0.49, 1.48); CagA: 0.96 (0.48, 1.92)	(1) (5)(6)(7)(10)
Lindkvist et al.	2008, Sweden	Nested case-control	ELISA	39/87	100/263	-	-	*H. pylori*: 1.25 (0.75, 2.09)	(1)(5)(7)(8)(9)(10)
Dou et al.	2008, China	Case-control	ELISA	54/85	64/136	29/85^c^	32/136^c^	*H. pylori*: 1.96 (1.12, 3.42); CagA & VacA: 2.10 (1.09, 4.06)	Not reported
Risch et al.	2010, the USA	Case-control	ELISA	80/373	120/690	55/373	108/690	*H. pylori*: 1.34 (0.94, 1.92); CagA: 0.83 (0.55, 1.24)	(1)(5)(7)(11)
Shimoyama et al.	2010, Japan	Case-control	ELISA	16/19	29/34	-	-	0.92 (0.19, 4.36)	Not reported
Qiao et al.	2012, China	Case-control	ELISA	41/63	44/100	19/63	7/100	*H. pylori*: 2.37 (1.24, 4.55); CagA: 5.74 (2.25, 14.66)	Not reported
Gawin et al.	2012, Poland	Case-control	ELISA, WB	121/139	146/177	116/139	150/177	*H. pylori*: 1.27 (0.64, 2.61); CagA: 0.90 (0.46, 1.73)	(1)(5)(7)(12)

^a^ The control group included 35 patients with colorectal cancer and 27 healthy volunteers.

^b^ Adjustments: (1) age, (2) month of blood draw, (3) completion with the dietary history, (4) intervention group assignment, (5) cigarette smoking, (6) years of education, (7) sex, (8) body mass index, (9) alcohol consumption, (10) time from baseline investigation to analysis, (11) ELISA plate number, (12) a family history of cancer.

^c^ These 2 ratios are for CagA+ & VacA+, and CagA- & VacA+, respectively.

### Quality assessment

The results of quality assessment according to NOS for case-control studies are shown in Table 2. All these studies reported that the diagnoses of all cases and controls were based on pathological and clinical records, and thus all studies got the two stars in the items of “adequate definition of cases” and “definition of controls”. All pancreatic cancer cases in each of the studies were declared to be diagnosed cases during a certain period, in certain medical centres, and thus the representativeness of cases was qualified for another star. *H. pylori* infection was identified by serological methods, so two more stars were assigned for “ascertain of exposure” and “same method to ascertain for cases and controls” to all studies. However, the same non-response rate between groups was not shown, or non-response rate was not mentioned in all studies, and thus all studies failed to win a star for “non-response rate”.

**Table 2:** Results of quality assessment by NOS for case-control studies.

Study	Selection				Comparability	Exposure			Scores
	Adequate definition of cases	Representative-ness of cases	Selection of controls	Definition of controls	Control for important factor ^a^	Ascertainment of Exposure	Same method to ascertain for cases and controls	Non-response rate	
Raderer et al.	☆	☆	-	☆	-	☆	☆	-	5
Stolzenberg et al.	☆	☆	☆	☆	☆☆	☆	☆	-	8
Dou et al.	☆	☆	-	☆	-	☆	☆	-	5
Lindkvist et al.	☆	☆	☆	☆	☆☆	☆	☆	-	8
de Martel et al.	☆	☆	☆	☆	☆☆	☆	☆	-	8
Risch et al.	☆	☆	☆	☆	☆☆	☆	☆	-	8
Shimoyama et al.	☆	☆	-	☆	-	☆	☆	-	5
Gawin et al.	☆	☆	-	☆	-	☆	☆	-	5
Qiao et al.	☆	☆	-	☆	-	☆	☆	-	5

^a^ A maximum of 2 stars can be allotted in this category, one for Age, the other for other controlled factors (gender, smoking and so on).

Overall, the scores of included studies ranged from five to eight stars, while four of them were defined high-quality [20,21,36,37]. 

### Overall meta-analysis

Generally speaking, the overall *H. pylori* positive rate in pancreatic cancer group (51.8%, 561 of 1083) was higher than that in control group (43.6%, 851 of 1950).

**Figure 2:**
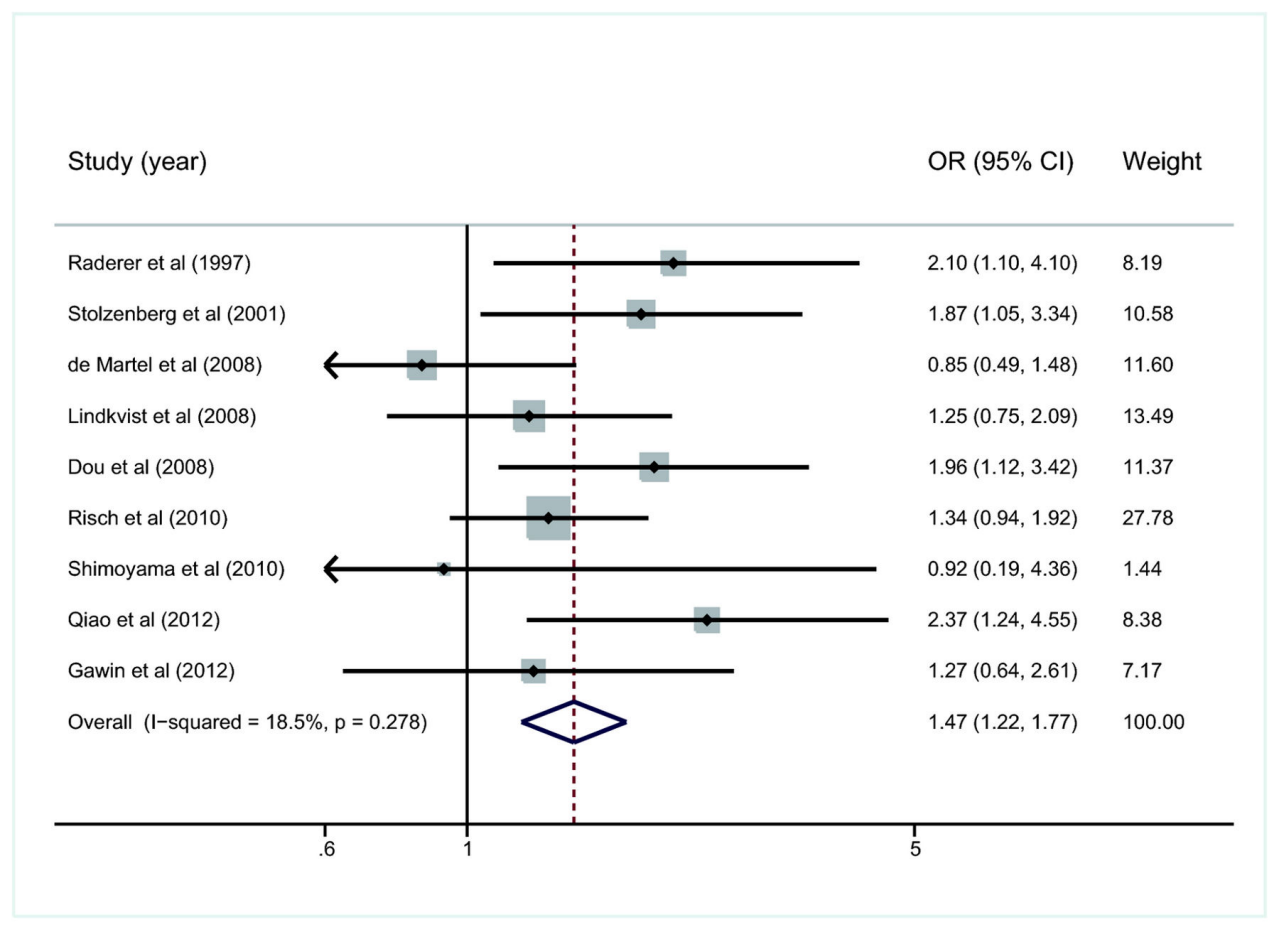
Overall meta-analysis of all the studies included for the association of *H. pylori* infection with pancreatic cancer. OR, odds ratio. CI, confidence interval.

The test for heterogeneity was not significant (*I* ^2^=18.5%, *p*=0.278), suggesting low heterogeneity among these studies. So we chose the fixed effect model when performing statistical analysis. The summary OR and 95%CI of the overall meta-analysis of all included studies were 1.47 and 1.22-1.77, respectively (Figure 2).

During the sensitive analysis, exclusion of any study did not influence the direction and magnitude of the cumulative estimates substantially (Table 3), revealing a relatively low sensitivity.

**Table 3:** Sensitivity analysis after each study was excluded by turns.

Study omitted	OR (95%CI) for remainders	Heterogeneity
Raderer *et al.* (1997)	1.43 (1.14, 1.79)	Insignificant (*I* ^2^=18.4%, *P*=0.284)
Stolzenberg *et al.* (2001)	1.44 (1.14, 1.83)	Insignificant (*I* ^2^=22.8%, *P*=0.248)
de Martel *et al.* (2008)	1.58 (1.29, 1.93)	Insignificant (*I* ^2^=0.0%, *P*=0.592)
Lindkvist *et al.* (2008)	1.53 (1.20, 1.95)	Insignificant (*I* ^2^=25.3%, *P*=0.227)
Dou *et al.* (2008)	1.43 (1.13, 1.80)	Insignificant (*I* ^2^=19.1%, *P*=0.278)
Risch *et al.* (2009)	1.52 (1.18, 1.99)	Insignificant (*I* ^2^=26.0%, *P*=0.221)
Shimoyama *et al.* (2010)	1.50 (1.20, 1.88)	Insignificant (*I* ^2^=26.1%, *P*=0.221)
Qiao *et al.* (2012)	1.41 (1.14, 1.73)	Insignificant (*I* ^2^=7.2%, *P*=0.375)
Gawin *et al.* (2012)	1.51 (1.19, 1.92)	Insignificant (*I* ^2^=18.5%, *P*=0.278)

### Sub-analysis of high-quality research

**Figure 3:**
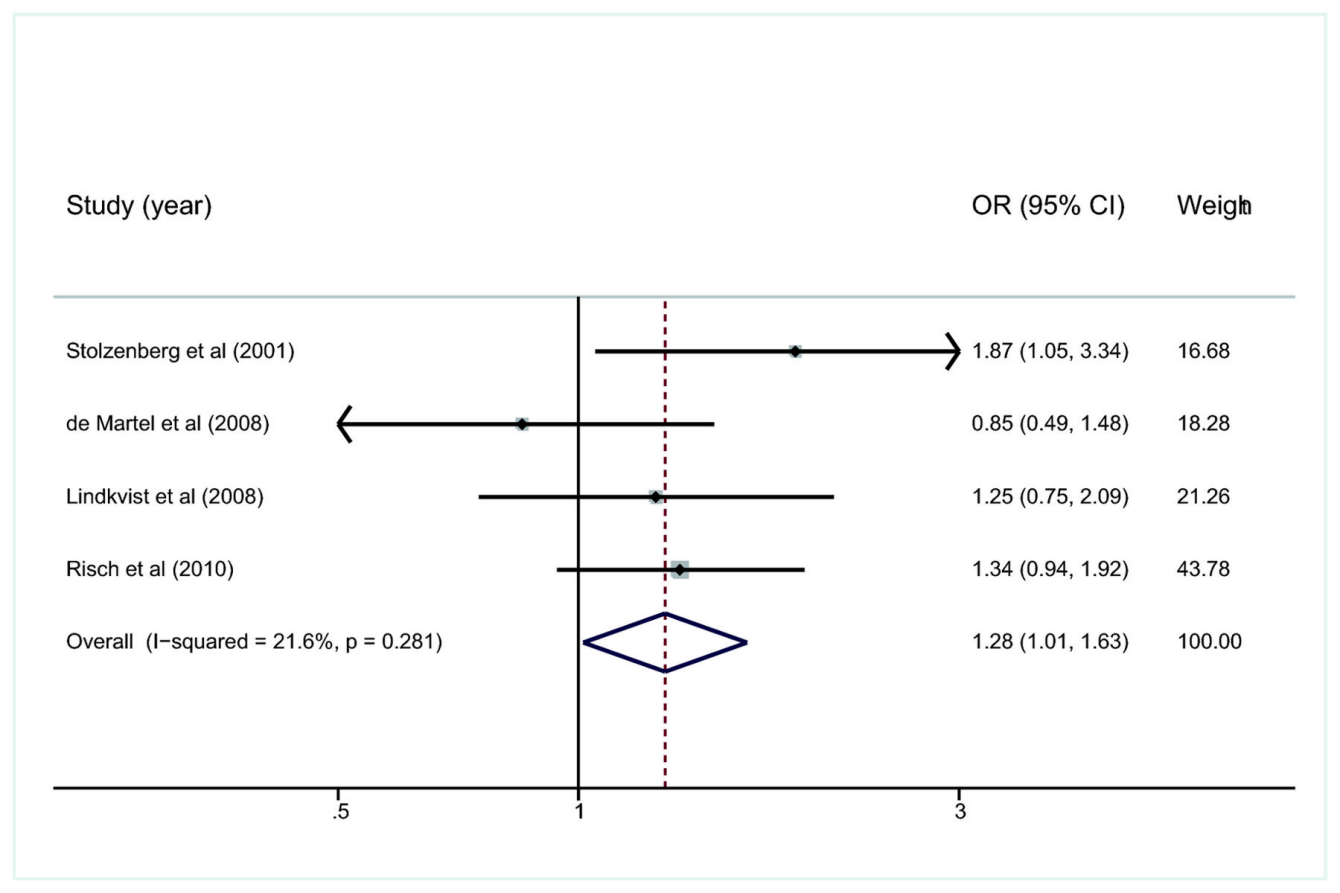
Meta-analysis of the four high-quality studies for the association of *H. pylori* infection with pancreatic cancer. OR, odds ratio; CI, confidence interval.

Sub-analysis involving the four high-quality studies is shown in Figure 3. Researchers of these studies also matched the controls to cancer cases in order to avoid confounding from age, gender and some other factors. The summary OR and 95%CI were 1.28 and 1.01-1.63, respectively, with significant heterogeneity (*I* ^2^=21.6%, *p*=0.281).

### Sub-analysis of studies detecting CagA

**Figure 4:**
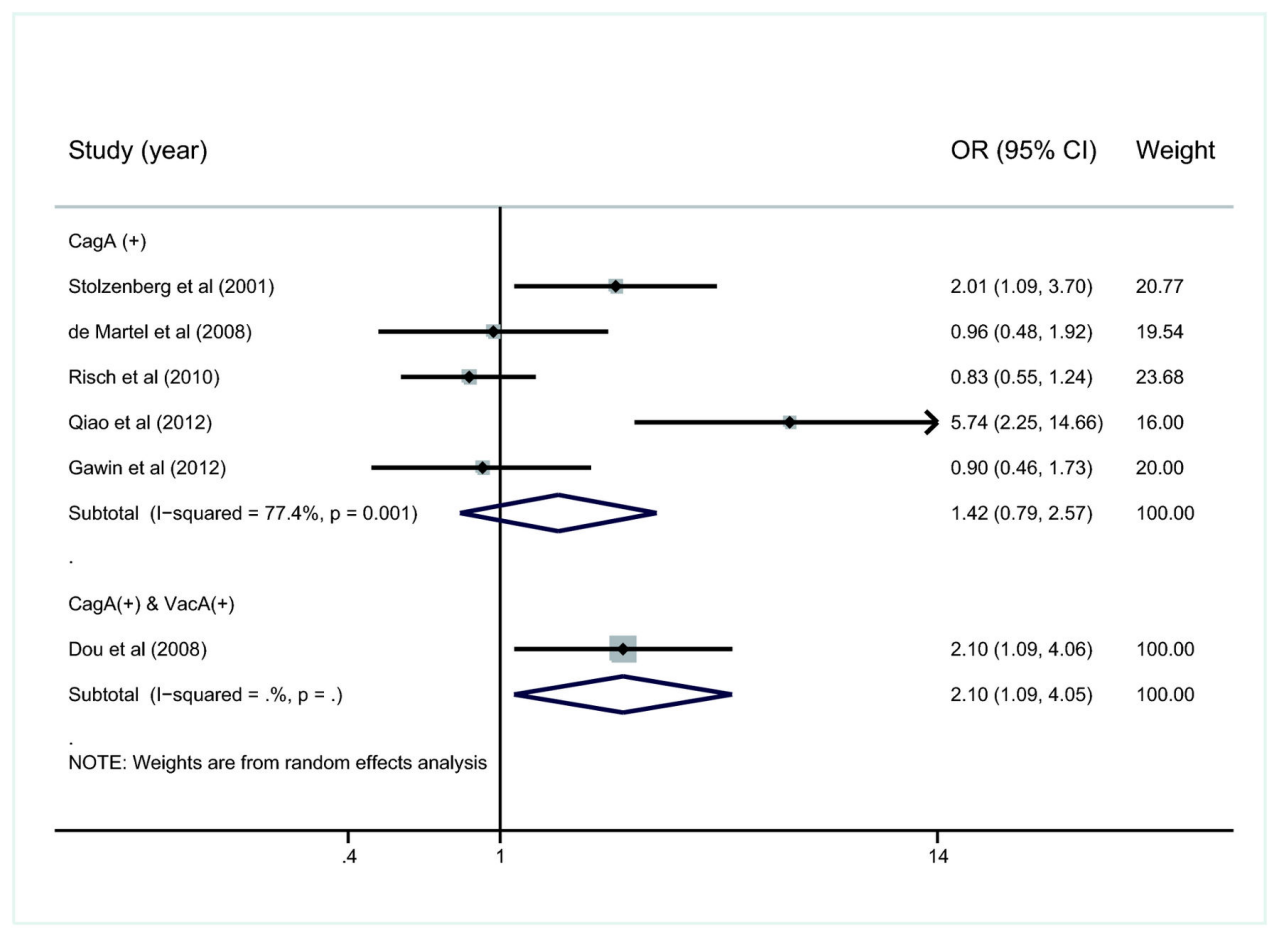
Meta-analysis of studies for the association of infection with CagA+ *versus* CagA- *H. pylori* strains and pancreatic cancer. OR, odds ratio; CI, confidence interval.

Among the six studies examining the infection rate of Cag+ *H. pylori* (including one study detecting CagA and VacA), the prevalence of CagA+ *H. pylori* ranged from 14.7% to 60.3% in pancreatic cancer patients, and from 7% to 84.7% in the controls. Three studies reported CagA+ *H. pylori* strains served as a risk factor of pancreatic cancer compared with CagA- strains, whereas the other three did not. The summary OR and 95%CI of the five studies detecting CagA+ only were 1.42 and 0.79 to 2.57, respectively, with significant heterogeneity (*I* ^2^=77.4%, *P*=0.001) (Figure 4). In addition, summary OR and 95%CI of 3 high-quality studies [20,36,37] were 1.14 and 0.66-1.97 respectively, with significant heterogeneity (*I* ^2^=64.8%) (figure was not shown). The only study that tested CagA and VacA indicated that *H. pylori* strains infection indicated that *H. pylori* strains harboring both CagA and VacA may serve as a risk factor for pancreatic cancer [23].

### Sub-analysis in relation to geographic regions

**Figure 5:**
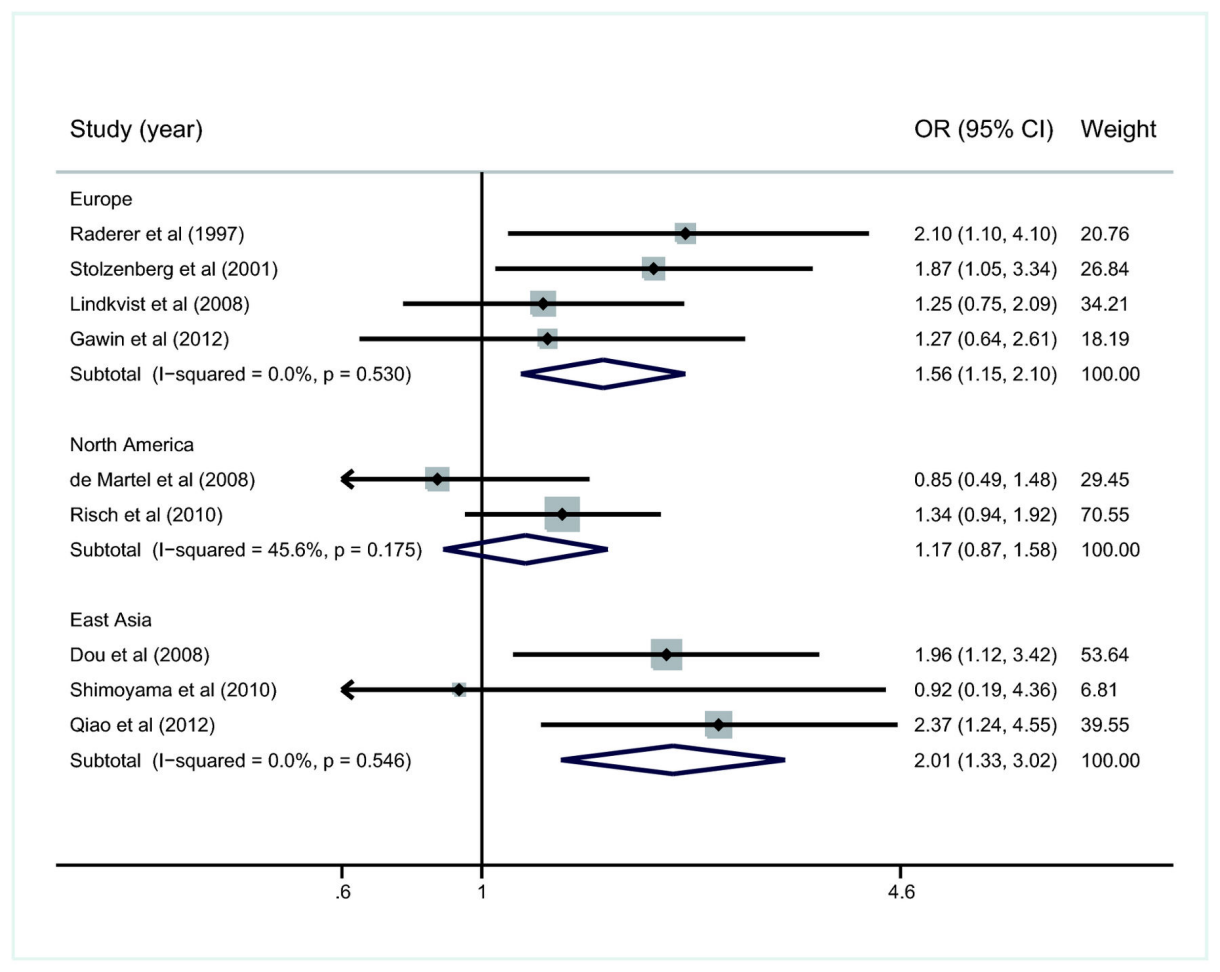
Subgroup analysis stratified by geographic distribution for the association of *H. pylori* infection with pancreatic cancer. OR, odds ratio; CI, confidence interval.

All nine studies were divided into three groups, Europe, North America, and East Asia. The summary ORs were 1.56 (95%CI: 1.15-2.10, *I* ^2^=0.0%), 1.17 (95%CI: 0.87-1.58, *I* ^2^=45.6%) and 2.01 (95%CI: 1.33-3.02, *I* ^2^=0.0%), respectively, in these regions (Figure 5).

### Publication bias

**Figure 6:**
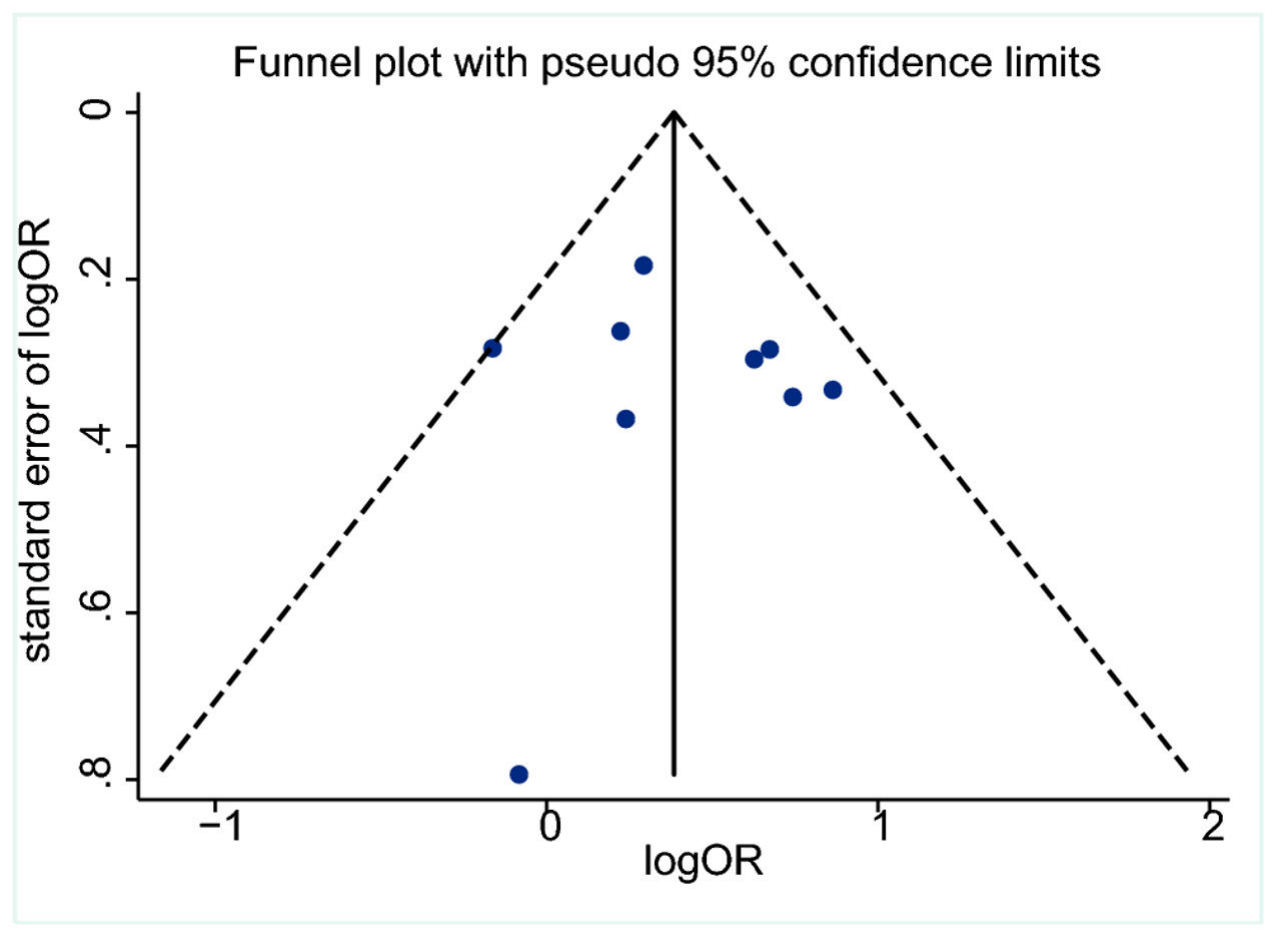
Funnel plot to detect publication bias.

The funnel plot manifested as an symmetrical appearance (Figure 6), and the *P* values for Begg’s test and Egger’ s test were 0.602 and 0.797 (continuity corrected), respectively, suggesting that there was no significant publication bias in our meta-analysis.

## Discussion

The association of *H. pylori* infection and pancreatic cancer development has long been investigated with controversial results. In this meta-analysis, we tried our best to include all available studies in this field, and found that based on the pooled data from all eligible studies, *H. pylori* seropositive rate was significantly higher in pancreatic cancer patients than in controls, indicating that *H. pylori* infection may serve as a risk factor for the development of pancreatic cancer. However, the summary OR and its 95%CI were just above 1 (1.47 and 1.22 to 1.77, respectively), suggesting that this positive association is nearly borderline significant and may have been biased by some confounders introduced by those non-high-quality studies [19,22-24,38]. Therefore, we conducted a sub-analysis which included only high-quality studies. The results still support a positive association between *H. pylori* and pancreatic cancer, although the pooled OR and corresponding 95%CI were both closer to the borderline value 1 (1.28 and 1.01-1.63, respectively).

CagA protein is the most studied virulence-associated factor of *H. pylori* [39]. Previous studies have demonstrated that CagA+ strains of *H. pylori* cause more severe gastritis inflammation, and are associated with a higher risk of developing intestinal metaplasia and thus intestinal-type gastric cancer [40,41]. However, in our meta-analysis, we found that CagA+ strains were not associated with the development of pancreatic cancer, indicating that CagA+ strains may not play a critical role in the development of pancreatic cancer. In other words, patients infected with CagA+ *H. pylori* are not at a higher risk of developing pancreatic cancer than those infected with CagA- strains. However, three of the six studies concerning CagA [20,22,23] detected CagA in *H. pylori* positive subjects only, which may have underestimated in the number of CagA+ cases and controls. Moreover, it has been reported that as time passes by, CagA antibodies seroreversion occurs to a substantially lower ratio than *H. pylori* antibodies seroreversion [42,43], and thus patients with a validated history of infection with CagA+ strains are *H. pylori* seronegativity but CagA seropositivity [37,44]. Therefore, the total number of subjects infected with CagA+ strains might have been underestimated in cases and controls, which might lead to a false negative conclusions. VacA is another important virulence factor in the pathogenesis of *H. pylori*-related diseases [45] which has been found to have immunosuppressive activity, leading to prolonged inflammatory reaction [46,47]. Only one study indicated that CagA+ and VacA+ strains increased pancreatic cancer risk by 2.10-fold [23], which needs to be confirmed by more studies. Taking together, further investigation is required to clarify the association between CagA+/VacA+ stains of *H. pylori* and pancreatic cancer.

The prevalence of *H. pylori* infection varied from region to region as well as from race to race [48,49]. To minimize the influence of geographic and ethnic factors, we conducted subgroup analysis stratified by regional distribution. We found that *H. pylori* infection contributes to a higher incidence of pancreatic cancer in Europe and East Asia, but not in North America. The two researches in the North America group are both high-quality, and reported no association between *H. pylori* and pancreatic cancer [36,37]. We also assessed the the two high-quality researches in Europe [20,21], and the summary OR and 95%CI were 1.49 and 1.02-2.19, respectively; the lower limit was approximate to the dividing value 1 (figure not shown), suggesting that the observed positive association might be the result complicated with some unnoticed confounders. None of the studies in the East Asia group were high-quality. Therefore, more studies, especially well designed and strictly implemented ones are needed to validate the association in each continent.

So far, studies have failed to demonstrate the colonization and growth of *H. pylori* or inflammation triggered by *H. pylori* in the human pancreas [25,50]. Therefore, *H. pylori* infection may exert its effect, if any, in the development of pancreatic cancer through certain indirect pathophysiologic processes following the infection in the stomach or even the duodenum, rather than direct stimulation. At least two hypothetical models have been proposed to explain the role of *H. pylori* in pancreatic carcinogenesis. One is the hypothetical pathway involving gastric antral colonization of *H. pylori*, hyperchlorhydria, enhanced release of secretin, proportional elevation in basal pancreatic bicarbonate output and pancreatic hyperplasia with accelerated metabolism and DNA synthesis, probably associated with a greater susceptibility to carcinogens [51]. The other plausible hypothesis involves *H. pylori* growth in the gastric corpus mucosa, atrophic gastritis, hypochlorhydria [52], which results in bacteria overgrowth and increased production of bacterially catalyzed N-nitrosamines, and transportation of these endogenous carcinogens to the host pancreas via bloodstream [53,54].

A few limitations exist in our meta-analysis. First, because of the lack of ‘gold standard’ bacteria culture to identify *H. pylori*, the positive association between *H. pylori* infection and pancreatic cancer may be due to cross reaction of *H. pylori* serological test with other *Helicobacter* species colonized in the human biliary tract and pancreas, such as *H. bilis*, *H. hepaticus* [50,55]. Second, a notable disadvantage of serological test is its disability of distinguishing between previous and active infection of *H. pylori* [56], because serological tests detects only antibodies to *H. pylori* other than *H. pylori* antigens, and thus a serological positive result may occur in either currently infected patients or patients who have received successful eradication therapy [57]. Third, all studies included in our meta-analysis are with a case-control design that is more susceptible to bias than prospective cohort studies, and this weakness can only be overcome by well-designed prospective studies, in order to draw a more convincing conclusion. Finally, it has been suggested that dietary intake of certain food, such as red and high-temperature cooked meat, and genetic variations in inflammation-related genes (such as COX-2) and susceptibility loci at chromosomes (such as 13q22.1, 21q21.3, 5p13.1) might be positively linked with pancreatic cancer [6,7,58,59], however, none of the eligible studies adjusted for these dietary or genetic factors. Therefore, future studies should also take these factors into account. Moreover, studies exploring the effects of virulence factors of *H. pylori*, the interactions of genetic polymorphisms and *H. pylori* infection, and the individual susceptibility of pancreatic cancer to *H. pylori* infection are also required to elucidate the role of *H. pylori* infection in the development of pancreatic cancer.

In conclusion, overall, *H. pylori* infection is significantly, albeit weakly, associated with pancreatic cancer. The association is prominent in Europe and East Asia, but not in North America. CagA+ *H. pylori* strains appear not to be associated with pancreatic cancer. More basic and clinical studies are required to further explore and validate the association between *H. pylori* infection and pancreatic cancer development.

## Supporting Information

Checklist S1 **PRISMA 2009 Checklist.**
(DOC)
